# Effectiveness of the Active School quasi-experimental cluster-controlled trial: Impact on objectively measured physical activity in Swiss secondary school students after 12 months

**DOI:** 10.1186/s12889-026-28690-6

**Published:** 2026-08-05

**Authors:** Marion Gasser, Andrea-Maria Nadenbousch, Fabienne Egger, Mario Kamer, Stefan Valkanover, Mirko Schmidt

**Affiliations:** 1 Institute of Sport Science, University of Bern, Bremgartenstrasse 145, Bern, 3012 Switzerland; 2 Institute for Primary Education, Bern University of Teacher Education, Fabrikstrasse 8, Bern, 3012 Switzerland; 3 Institute for Lower Secondary Education, Bern University of Teacher Education, Fabrikstrasse 8, Bern, 3012 Switzerland; 4 Centre of Subject Didactics, Bern University of Teacher Education, Fabrikstrasse 8, Bern, 3012 Switzerland

**Keywords:** Physical activity, Comprehensive school physical activity program, Secondary school, Adolescents, Swiss setting

## Abstract

**Background:**

Swiss adolescents exhibit insufficient physical activity (PA), with most not meeting daily and school-based PA recommendations, and girls being less active than boys. Comprehensive school programs promoting PA across multiple school segments, supported by trained school-based teachers, are considered a promising strategy for sustainably increasing PA. Yet, evidence of their effectiveness—particularly regarding longitudinal, segment-specific changes—remains limited. This study examines the 12-month effectiveness of *Active School*, a comprehensive PA promotion program allowing schools to autonomously decide which components and goals to implement across different school segments, in Swiss secondary schools, focusing on objectively measured PA across school segments.

**Methods:**

A quasi-experimental cluster-controlled trial was conducted in 12 self-selected, voluntarily participating secondary schools in the Canton of Bern, Switzerland (six experimental, six wait-list control schools continuing usual practice without PA promotion). In total, 703 students were assessed (mean age 13 years; 49.8% girls). PA in both groups was objectively measured using wrist-worn accelerometers at baseline and after 12 months of mid-term intervention implementation. The primary outcome was moderate-to-vigorous physical activity (MVPA); secondary outcomes were inactivity (IN), light PA (LPA), step counts, and the proportion of students meeting PA-recommendations during school time (≥ 30 min MVPA) and across the whole day (≥ 60 min MVPA). Outcomes were analyzed across school segments (classtime, recess, physical education), the entire school time, and the whole day. Intervention effects were estimated using linear mixed-effects models.

**Results:**

No significant intervention effects were observed for MVPA, IN, or LPA across any segments, nor for the proportion of students meeting PA recommendations. However, a significant positive intervention effect emerged for step counts during classtime (adjusted difference in change: 36 steps/hour; 95% CI 15.8 to 56.38, *d* = 0.29), favoring experimental schools. Exploratory analyses indicated that this effect was driven by girls: those in experimental schools showed a significant increase in their classtime step counts (*p* < .001, *d* = 0.56), whereas no change was observed among boys (*p* = .651, *d* = 0.06).

**Conclusions:**

After 12 months, *Active School* did not improve MVPA or reduce IN, but showed positive effects on step counts during classtime. Given the overall small intervention effects future research should examine implementation fidelity and identify which intervention components may strengthen intervention effectiveness.

**Trial registration:**

German Clinical Trials Register (DRKS00033362), registered January 25, 2024. Retrospectively registered.

**Supplementary Information:**

The online version contains supplementary material available at https://doi.org/10.1186/s12889-026-28690-6.

## Background

A substantial proportion of adolescents worldwide fail to achieve the recommended 60 min of moderate-to-vigorous physical activity (MVPA) per day, with girls consistently showing lower activity levels than boys [1]. Similar patterns are also observed in Switzerland, where more than half of adolescents fail to achieve daily PA recommendations and spend excessive time in inactive behavior [2–5]. Given the benefits of PA and the detrimental effects of inactivity (IN) on physical and mental health [6–9], this trend is concerning. These patterns highlight the need for strategies promoting active daily routines among Swiss adolescents, including increasing MVPA and reducing IN [10].

Schools represent a key setting for PA promotion [11], as young people spend a substantial proportion of their day in this environment, and regular PA offers benefits beyond health, including improvements in memory, concentration, and academic achievement [12–14]. Accordingly, schools are encouraged to contribute half of the daily PA recommendation by providing at least 30 min of MVPA per day during school time [15]. Consequently, school-based PA programs have been widely implemented internationally in recent years [16]. Evidence suggests that these programs can be effective, with some studies demonstrating increases in MVPA [17, 18], or reductions in IN [19]. However, reported effects are generally small (SMD ≈ 0.24), and sustaining these changes remains a major challenge [20, 21]. Many interventions rely on researcher support, and effects frequently diminish once this support is withdrawn [22]. This highlights the importance of embedding PA promotion within school systems and strengthening internal leadership to support long-term sustainability beyond externally funded research periods.

Comprehensive school approaches have increasingly been discussed as the most promising strategy for promoting sustainable changes in adolescents’ PA behavior [23–25]. These approaches emphasize school-specific and individually tailored PA goals, promote PA across multiple school segments, and are coordinated through internal school leadership [26]. They involve the entire school community, rather than targeting individual teachers or isolated activities [27]. By embedding PA promotion within school-improvement processes and enabling context-specific adaptation over time [22], comprehensive approaches can establish sustainable structures, with international evidence demonstrating maintained effects of PA promotion at one-year follow-up and beyond [28–30].

Switzerland currently lacks comprehensive PA programs, with existing initiatives largely limited to the primary school level [31–33]. To address this gap, *Active School* was developed as the first comprehensive PA approach in Swiss secondary schools [34]. It targets multiple school segments, such as classtime, recess, and physical education (PE), as expanding PA opportunities is considered central to increasing students’ activity levels [35]. Given the importance of internal leadership for successful implementation [36], Physical Activity Leaders (PALs)— teachers within the school—coordinate and adapt school-specific PA goals based on needs assessments of students and teachers. As this role extends beyond teachers’ traditional responsibilities [37], PALs receive professional development (PD) training, a distinctive feature of *Active School*.

In evaluations of comprehensive school-based PA programs, several components warrant particular attention due to their complex and multilayered nature [38, 39]. A key consideration is that comprehensive approaches target PA across multiple school segments, making segment-specific outcomes more informative than whole-day measures for identifying where intervention effects occur [40, 41]. While a growing body of research has identified which school segments contribute most to adolescents’ overall PA levels, this evidence is largely based on cross-sectional analyses, while longitudinal PA changes are scarce [42, 43]. Longitudinal evidence suggests that PA trajectories differ across school segments, with more stable patterns in structured contexts such as classroom time [43], whereas increases in recess or PE are more difficult to achieve and sustain, even within PA interventions [42].

Comprehensive school approaches also appear particularly well-suited to increasing PA among less active and at-risk groups, including adolescent girls [44–46]. Girls and boys differ in preferences, motivations, and perceived barriers to PA, indicating uniform strategies are unlikely to be effective across genders [47, 48]. Comprehensive approaches have the potential to address this challenge by incorporating needs assessments and student involvement, enabling programs to respond to gender-specific interests and preferences [26]. Consequently, evaluations of such programs should explicitly examine gender-specific effects, as a recent systematic review highlights that insufficient attention to gender in implementation and evaluation may limit conclusions about what works for both girls and boys [49].

Since comprehensive school approaches allow substantial autonomy in implementation, adoption of PA promotion strategies may differ across schools or even classes [50, 51]. Such variability may arise from differences in school-level conditions (e.g., organizational support or infrastructure) as well as class-level factors, including teachers’ motivation, competence, and perceived barriers to implementation [36, 52, 53]. Examining such heterogeneity, including identifying schools or classes that respond more strongly to the intervention, can help generate theoretically informed assumptions regarding the contextual and implementation-related factors shaping effectiveness [36].

Therefore, the present study aimed to evaluate the effectiveness of *Active School* after 12 months, focusing on objectively measured PA. Specifically, it examined (1) changes in PA levels across different school segments and across the whole day, and (2) intervention effects on adherence to school-based and daily PA recommendations. In addition (3), exploratory analyses investigated potential gender-specific differences and heterogeneity of intervention-related changes across schools and classes to generate hypotheses for future implementation-focused research.

## Methods

### Study design, setting and school recruitment

*Active School* was designed as a cluster-randomized controlled trial [34]. However, random allocation was partially constrained, as three schools expressed interest in the program but opted to participate as wait-list control schools due to limited temporal and organizational capacities. As a result, the study followed a quasi-experimental cluster-controlled design.

In total twelve secondary schools in the canton of Bern, Switzerland, participated in *Active School* (six experimental, six wait-list control). Assessments were conducted in both groups at baseline and at the 12-month mid-intervention assessment (post assessment). Schools were recruited via newsletters from the Bern University of Teacher Education and emails distributed through the official cantonal education authority to all eligible secondary schools in the Canton of Bern. Eligible schools were public or semi-public, non-specialized schools (grades 7–9) and not involved in major PA promotion initiatives. In Switzerland, semi-public schools are privately managed schools that receive partial public funding, while non-specialized schools are not specifically focused on sports, arts, or special educational needs. Participation was voluntary following these invitations, which may have increased the risk of convenience sampling and selection bias.

### Participants and sample size

A priori sample size calculations were conducted using the R package *powerlmm* [54]. The calculation was based on the following assumptions: A standardized effect size (Cohen’s *d*) of 0.25 for daily MVPA, based on the SCORES comprehensive school-based intervention in adolescents, reporting an adjusted between-group difference of 5.7 min/day [28], intraclass correlation coefficients (ICCs) of 0.01 at the school level [55] and 0.70 at the within-student level [56] and an anticipated attrition rate of 35% [57]. Based on these parameters, simulation-based analyses for a cluster-randomized design with six schools per arm and 45 students per school yielded a statistical power of 0.81 (α = 0.05, two-sided). Accordingly, a minimum sample size of 540 students was required.

All 7th-grade students from the 12 recruited schools were invited to participate. Eligibility required the physical ability to complete PA assessments and written parental consent. At baseline, 703 students (345 experimental; 358 wait-list control) provided consent and were assessed, while 219 declined. Although reasons for non-participation were not systematically assessed, this may indicate a potential self-selection of more motivated students, representing a possible source of selection bias. At post assessment, 39 newly enrolled students were included. These students either decided to participate in the study after baseline assessment (*n* = 8) or had joined the class due to relocation or grade repetition (*n* = 31). All participants provided written informed consent prior to participation.

**Fig. 1:**
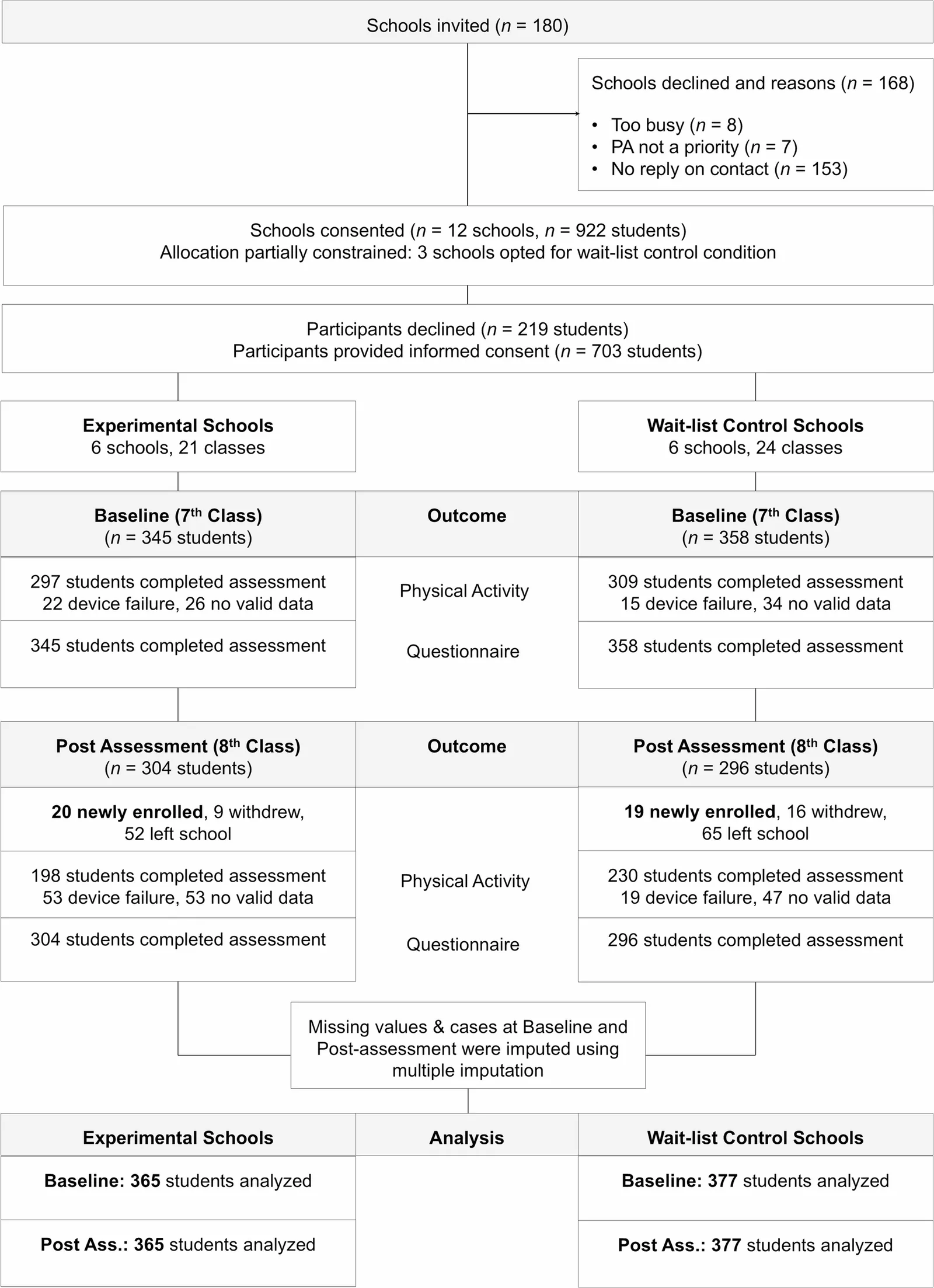
CONSORT-style flow diagram showing school, class, and student participation at baseline and 12-month post assessment. The completed CONSORT checklist and additional information on data handling are provided in Additional file 1

During the study, data loss occurred due to accelerometer malfunction, invalid wear time, school transfers, or voluntary withdrawal (see Fig. 1, Additional file 1). Little’s MCAR test [58] was not significant, χ²(19306) = 1911.08, *p* > .999, indicating completely random missingness. However, as the proportion of missing data exceeded 5%, missing values and incomplete cases at both baseline and post assessment were handled using multiple imputation [59]. The final analytical sample comprised 742 students at baseline and post assessment.

### Intervention

*Active School* was developed based on the international Comprehensive School Physical Activity Program (CSPAP) framework [26]. Detailed descriptions of the intervention are available in the study protocol [34], which was published one year prior to the first outcome analyses. In addition, the TIDieR checklist is provided in Additional file 1. Schools defined individualized PA promotion goals based on teacher and student needs assessments, targeting at least one—and typically multiple—of five components: PA during school, PE, PA before and after school, staff involvement, and family and community engagement.

**Fig. 2:**
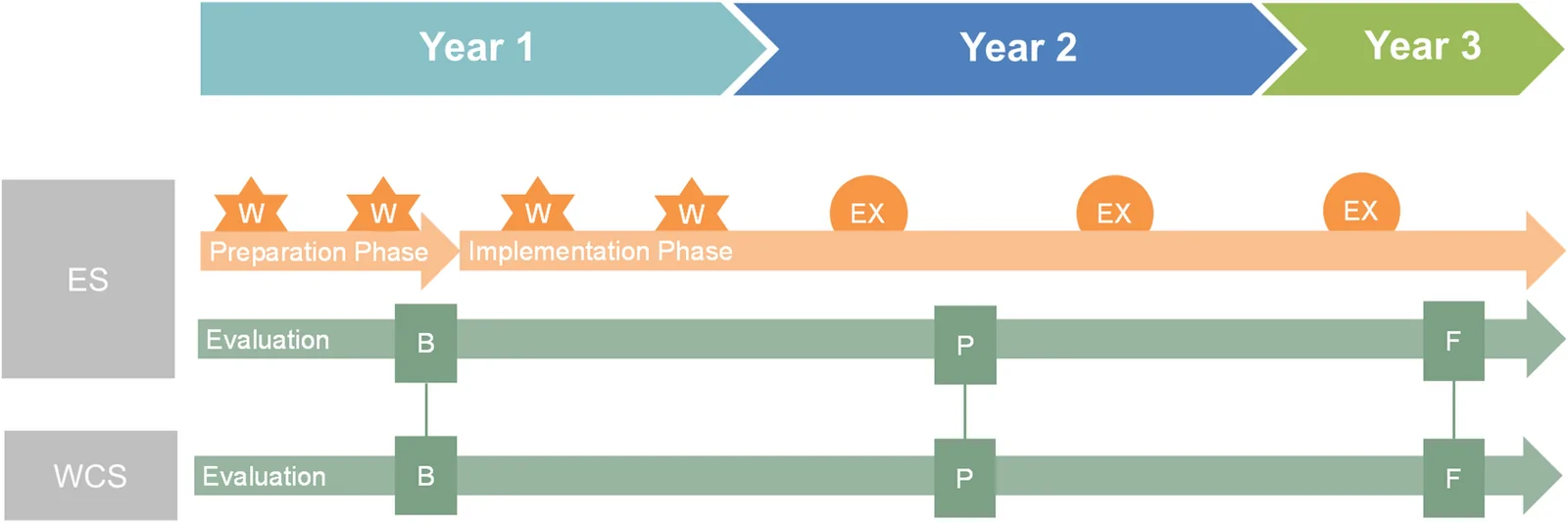
Intervention and evaluation timeline. ES = experimental schools, WCS = wait-list control schools, W = workshops, EX = exchange meetings, B = baseline, P = post assessment, F = follow-up assessment

Experimental schools implemented their PA promotion goals over a 2.5-year period comprising a six-month preparation phase and a two-year implementation phase, supported by structured implementation tools. Two PALs per school, were responsible for planning, implementing, and evaluating school-specific PA measures and for engaging the wider school community to embed PA promotion across the school. In five schools, PALs were exclusively PE teachers; in one school, one PAL was a non-PE teacher. PALs participated in a research team–led PD training based on the Quality Implementation Framework [60], consisting of workshops and exchange meetings focused on implementation strategies [61], including needs assessment, planning and adaptation, capacity building, the integration of PA into daily school routines, and evaluation. Examples of PA-related measures addressed during the PD training included the structured integration of active breaks and physically active learning activities into regular classroom routines, the provision of activity equipment and optional organized activity zones to promote PA during recess, and strategies to maximize students’ active time during PE lessons, such as minimizing transition time. The specific measures ultimately implemented were selected by the PALs and schools based on needs identified through the structured needs assessment process and adapted to local capacities and contextual conditions. These workshops and exchange meetings brought together PALs from all experimental schools, thereby fostering inter-school exchange. The preparation phase included two half-day workshops held at the university on planning and capacity building, followed by an implementation phase supported by two additional workshops and three exchange meetings addressing ongoing implementation processes. PALs also received continuous mentoring and access to technical resources. The present evaluation assessed short-term intervention effects after 12 months by comparing baseline and post assessment PA outcomes (see Fig. 2). A comprehensive process evaluation examining implementation dose, reach and feasibility and the specific PA measures implemented across schools is currently ongoing and was not part of the present study.

The wait-list control schools continued their regular school routines, including mandatory PE lessons as part of the Swiss school curriculum, but without additional school-wide PA promotion strategies implemented across other school segments. After study completion, control schools were offered access to all project materials and optional PAL training.

### Measures

#### Students characteristics

Anthropometric measures (height, weight, age, gender) and demographic indicators (family health climate, socioeconomic status) were collected. Height was obtained to the nearest 0.1 cm with a portable stadiometer (Anthrofex, NutriActiva, Germany), and weight was recorded to the nearest 0.1 kg using a digital scale (EBS002K, Esperanza). Body mass index (BMI) was computed using age- and gender-specific references [62]. Family health climate was assessed using the Family Health Climate Scale [63] and socioeconomic status via the Family Affluence Scale III [64].

#### Primary outcomes

MVPA was assessed using GENEActiv accelerometers (ActivInsights Ltd., UK) worn on the non-dominant wrist, which have demonstrated validity for estimating PA intensities in adolescents [65]. Accelerometers were initialized at 87.5 Hz, with raw signals aggregated into 1-second epochs and processed using the R package GGIR [66]. Devices were worn for nine consecutive days; the first and last day and weekend days were excluded to minimize reactivity and to focus on school days, yielding five complete school days for analysis (Monday to Friday) [67]. A school day was defined as valid if ≥ 10 h of wake wear time (excluding sleep) were recorded [56]. For students with ≥ 3 valid school days, daily averages were calculated; otherwise, data were treated as missing and imputed according to recommended procedures (see Additional file 1) [68]. Established non-dominant wrist cut-points were used to classify MVPA [65], which was expressed as relative percentages (%) within each analyzed time segment to account for differences in wear time [69]. Primary outcomes included MVPA during classtime (i.e., regular academic lessons), recess, PE, and entire school time (excluding lunch breaks, as students in our sample left school during lunchtime), and the whole day (excluding sleep). Segmentation of PA was performed using individual class timetables, with timetable-specific timestamps and segment labels imported into the GGIR processing pipeline to segment raw accelerometer data into the respective school-day periods.

#### Secondary outcomes

Secondary outcomes included inactivity (IN) and light physical activity (LPA), expressed as segment-specific time based on validated GENEActiv intensity cut-points [65]. IN refers to time spent in the lowest accelerometer-derived intensity category, including sitting, lying, and other minimal-movement states. Step counts were derived as an indicator of ambulatory PA using the Verisense algorithm implemented in GGIR [70] and expressed as counts per hour to account for differences in wear time. A previous study demonstrated acceptable validity of this wrist-worn step-count approach in young adult populations, including when using GENEActiv devices [71]. For analyses of guideline adherence, absolute minutes of MVPA were calculated. Guideline adherence was then assessed as the proportion of students meeting school-based (≥ 30 min MVPA) and daily (≥ 60 min MVPA) PA recommendations.

### Procedure

The sample consisted of two cohorts, with baseline assessment in summer 2023 or 2024 and post assessment 12 months later. Data collection at both baseline and post assessment was organized into two classroom visits. During the first visit, accelerometers were distributed, and anthropometric measurements were conducted using standardized procedures and instructions. During the second visit, nine days later, the accelerometers were collected, and students completed a paper–and–pencil questionnaire assessing students’ characteristics variables. Students were incentivized with small rewards for consistent device wear.

### Statistical analysis

All analyses were conducted in R (version 4.4.0). Multiply imputed datasets were analyzed separately and pooled using Rubin’s Rules [72]. Descriptive statistics were computed for all student characteristics, and independent-samples *t*-tests were used to compare the experimental and wait-list control schools at baseline. Attrition bias [73] was assessed via independent-sample *t*-tests comparing completers with baseline-only participants on gender, age, BMI, and PA levels. Sensitivity analyses using complete-case data yielded results comparable to those from multiply imputed analyses. A two-sided significance level of α < 0.05 was applied, with Holm–Bonferroni correction used where appropriate.

Primary and secondary outcomes were analyzed using linear mixed-effects models (LMMs) following the intention-to-treat principle. Null models were estimated to examine variance components across hierarchical levels and compared using likelihood-ratio χ²-tests. All final models retained a four-level structure (measurement occasions nested within students, classes, and schools) to reflect the hierarchical design [74]. Models included random intercepts for students, classes, and schools, and fixed effects for group (experimental vs. wait-list control), time (baseline vs. post assessment), and their interaction, which represented the primary test of intervention effects. Random slopes for time were included when they improved model fit. Gender was included as a covariate. In addition, planned moderator analyses (e.g., physical self-concept, motivation for exercise) were conducted as specified in the study protocol; however, as model fit was not improved, these interaction terms were not retained in the final models.

To limit inflation of type I error due to multiple hypothesis testing across related outcomes, familywise error rates were controlled using the Holm–Bonferroni procedure [74, 75]. Outcome families were defined a priori based on theoretical and conceptual grouping of PA constructs: MVPA outcomes constituted the primary outcome family, while secondary outcomes were grouped by activity type (IN, LPA, and step counts) across school-day segments. Binary outcomes for meeting school-based (≥ 30 min MVPA) and daily (≥ 60 min MVPA) PA recommendations were analyzed using linear probability mixed models with the same fixed- and random-effects structure [76].

Gender-specific effects were explored by extending the models with a group × time × gender interaction. As outlined in the study protocol, the trial was not powered to detect three-way interaction effects; therefore, these analyses were considered exploratory, although theoretically motivated by prior evidence of gender differences in PA [77]. When the interaction term reached statistical significance (two-sided α < 0.05, Holm–Bonferroni adjusted as in the primary analyses), stratified linear mixed models were estimated separately for boys and girls. For outcomes showing significant intervention effects, additional exploratory analyses were conducted within the experimental schools to examine potential heterogeneity across schools and classes. These analyses included the calculation of intraclass correlation coefficients (ICCs) of individual change scores, comparisons of change scores across units using analysis of variance, and adjusted within-unit pre–post changes with correction for multiple testing.

## Results

### Baseline characteristics and attrition analyses

At baseline, experimental and wait-list control schools did not differ in age, anthropometric indicators, socioeconomic status, or family health climate (all adjusted *p*s ≥ .07, |*d*| ≤ 0.20; Table 1). In addition, no significant baseline differences were observed for most PA outcomes (see baseline data in Table 2), except for MVPA during recess and the whole day, as well as step counts during entire school time and classtime, where the experimental schools showed lower baseline levels (all adjusted *p*s ≤ .035). Attrition analyses showed no significant differences between completers and baseline-only participants in age, BMI, daily MVPA, or gender distribution (all *p*s ≥ .143), indicating that attrition was unlikely to be selective.

**Table 1:** Baseline sample characteristics stratified by intervention condition (*M*,* SD*, *t*,* p*,* d*)

	**Overall**	**Experimental ** **Schools**	**Wait-list Control Schools**	**Group Differences**		
**Sample Characteristics**	(*N * = 742)	(*n* = 365)	(*n* = 377)	*t* (740)	*p*	*d*
Girls *n* (%)	370 (49.8%)	184 (50.4%)	186 (49.3%)			
Age (years)	13.28 (0.57)	13.22 (0.55)	13.33 (0.58)	2.55	0.07	0.20
Weight (kg)	54.36 (11.48)	53.75 (11.09)	54.95 (11.83)	1.42	0.32	0.10
Height (cm)	163.18 (7.78)	162.64 (7.83)	163.71 (7.70)	1.86	0.62	0.14
BMI (kg · m^− 2^)	20.29 (3.44)	20.21 (3.28)	20.37 (3.58)	0.64	0.99	0.05
Socioeconomic Status (0–13)	9.71 (1.81)	9.64 (1.86)	9.77 (1.76)	0.92	0.99	0.07
Family Health Climate (0–3)	1.93 (0.55)	1.95 (0.53)	1.91 (0.56)	-0.84	0.99	-0.07

*p* values are Holm–Bonferroni adjusted for multiple comparisons. *d* = Cohen’s *d*

**Table 2:** Changes in primary and secondary PA outcome measures and group differences after 12 months

	**Experimental, Mean (SD)**		**Wait-list Control, Mean (SD)**		**Adjusted Difference**	*P* Value	**ICC**
	**Baseline**	**Post**	**Baseline**	**Post**	**in Change (95% CI)**		
**Measures**	(*n* = 365)	(*n* = 365)	(*n* = 377)	(*n* = 377)			
Primary Outcome PA							
MVPA entire schooltime (%)	8.46 (2.89)	7.71 (2.30)	8.44 (2.74)	7.93 (2.59)	-0.28 [-0.78, 0.23]	0.566	0.02
MVPA classtime (%)	5.47 (2.17)	5.60 (1.88)	5.51 (2.25)	5.45 (2.24)	0.18 [-0.22, 0.59]	0.566	0.01
MVPA recess (%)	19.71 (9.05)	15.57 (6.56)	17.33 (7.64)	15.20 (6.10)	-2.17 [-3.46, -0.88]	**0.005**	0.02
MVPA PE (%)	30.49 (10.34)	26.03 (9.40)	31.25 (10.71)	28.61 (10.07)	-1.83 [-3.74, 0.09]	0.186	0.06
MVPA WD (%)	9.50 (2.95)	8.55 (2.21)	8.93 (2.63)	8.52 (2.52)	-0.50 [-0.99, -0.00]	0.192	0.03
Secondary Outcome PA							
IN entire schooltime (%)	71.80 (5.61)	73.07 (4.49)	71.84 (5.46)	73.14 (4.97)	0.08 [-0.78, 0.93]	0.862	0.03
IN classtime (%)	76.88 (5.02)	76.90 (4.17)	76.96 (5.13)	77.58 (4.77)	-0.55 [-1.40, 0.31]	0.630	0.01
IN recess (%)	47.79 (12.16)	53.37 (10.19)	49.50 (12.26)	53.24 (9.99)	2.32 [0.24, 4.41]	0.116	0.02
IN PE (%)	38.80 (14.06)	44.99 (13.63)	38.64 (13.73)	41.78 (13.57)	3.13 [0.43, 5.83]	0.115	0.03
IN WD (%)	70.75 (5.06)	72.00 (4.00)	71.32 (4.82)	72.10 (4.43)	0.42 [-0.42, 1.25]	0.652	0.03
LPA entire schooltime (%)	19.77 (3.28)	19.21 (2.88)	19.70 (3.32)	18.90 (3.00)	0.18 [-0.37, 0.72]	0.892	0.05
LPA classtime (%)	17.67 (3.30)	17.58 (2.92)	17.52 (3.34)	16.92 (3.07)	0.47 [-0.12, 1.06]	0.464	0.03
LPA recess (%)	32.57 (6.03)	31.02 (5.53)	33.22 (7.10)	31.49 (5.69)	-0.08 [-1.29, 1.12]	0.892	0.03
LPA PE (%)	30.76 (6.17)	28.87 (6.37)	30.04 (6.41)	29.55 (6.12)	-1.52 [-2.81, -0.22]	0.110	0.01
LPA WD (%)	19.75 (2.93)	19.46 (2.59)	19.73 (2.95)	19.35 (2.71)	0.08 [-0.44, 0.61]	0.892	0.02
Steps entire schooltime (counts/h)	666.73 (134.16)	673.32 (111.50)	693.52 (135.99)	671.78 (119.63)	24.01 [2.43, 45.60]	0.116	0.05
Steps classtime (counts/h)	524.10 (118.04)	560.56 (102.90)	545.12 (130.95)	542.21 (115.37)	36.09 [15.80, 56.38]	**0.005**	0.01
Steps recess (counts/h)	1518.20 (443.55)	1382.60 (369.90)	1505.00 (417.82)	1429.44 (411.57)	-76.40 [-151.70, -1.10]	0.141	0.02
Steps PE (counts/h)	1425.78 (348.44)	1382.20 (362.01)	1481.63 (339.06)	1435.28 (328.34)	1.05 [-66.90, 69.00]	0.976	0.01
Steps whole day (counts/h)	761.32 (147.10)	759.73 (122.71)	741.21 (128.81)	754.34 (120.43)	-14.86 [-36.96, 7.25]	0.376	0.06

	**Experimental**, *n* (%)		**Wait-list Control**, *n* (%)				
	**Baseline**	**Post**	**Baseline**	**Post**			
Meeting PA-recommendation							
30 min MVPA school	178 (48.8)	137 (37.5)	178 (47.2)	154 (40.8)	-0.05 [-0.15, 0.05]	0.622	0.03
60 min MVPA whole day	321 (87.9)	313 (85.8)	323 (85.7)	314 (83.3)	-0.00 [-0.06, 0.06]	0.972	0.02

*PA*  Physical activity, *PE*  Physical education, *WD*  Weekday, *ICC*  Intraclass correlation coefficient (school level)

*p* values refer to the group × time interaction effects and were Holm–Bonferroni adjusted for multiple comparisons. Bold values indicate statistical significance after adjustment. Percentage values represent the relative time spent in each activity intensity within the respective time segment

### Primary outcome analyses

**Fig. 3:**
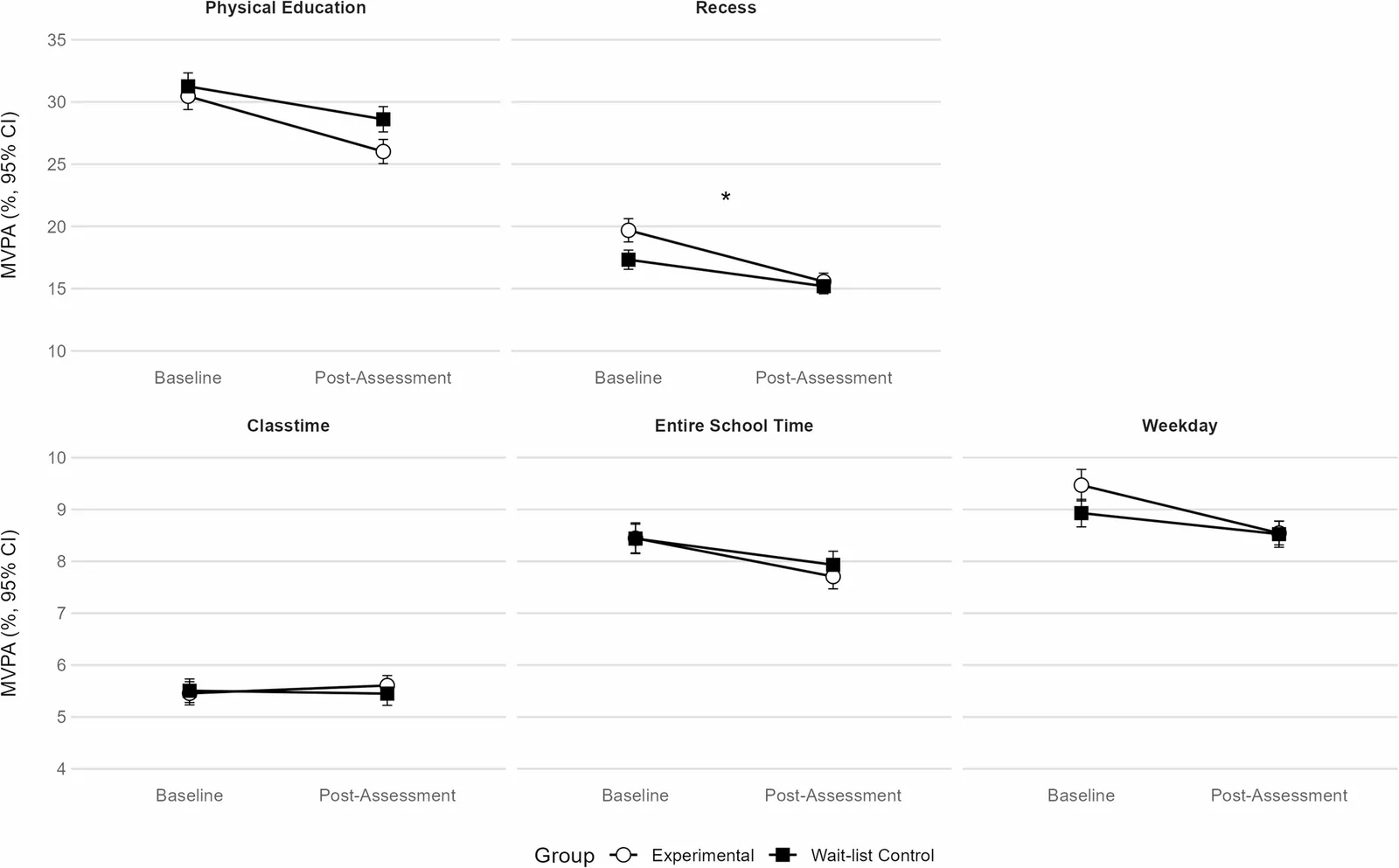
MVPA (%) at baseline and post-assessment stratified by group across different school-time segments (physical education, recess, classtime, entire school time, and weekday). Error bars represent 95% confidence intervals. *adjusted *p* < .05

No significant group × time interactions were observed for MVPA during the entire school time, classtime, PE, and whole day (all Holm-Bonferroni adjusted *p*s ≥ .186, see Table 2). MVPA declined significantly from baseline to post assessment in both groups during entire school time, PE, and the whole day (all Holm-Bonferroni adjusted within-group *p* ≤ .013), whereas classtime MVPA remained stable over time in both the experimental and wait-list control schools (all Holm-Bonferroni adjusted *p*s ≥ .333). These overall declines in MVPA are also visually apparent in Fig. 3.

A statistically significant group × time interaction was observed only for MVPA during recess (Holm-Bonferroni adjusted *p* = .005). MVPA was higher in the experimental schools at baseline (*t* = 3.84, *p* < .001, *d* = 0.29), whereas no group difference was observed at post assessment (*t* = − 0.77, *p* = .439, *d* = − 0.06), indicating a smaller decline in the wait-list control schools.

### Secondary outcome analyses

No significant group × time interactions were observed for IN or LPA across any school or whole day segments (all Holm-Bonferroni adjusted *p*s ≥ .110).

For classtime step counts, a significant group × time interaction was observed (*b* = 36.75, *SE* = 10.34, *t* = 3.54, Holm–Bonferroni adjusted *p* = .005, Cohen’s *d* = 0.29). Compared with wait-list control schools, step counts were lower in the experimental schools at baseline (*t* = 2.29, *p* = .022, *d* = 0.17), but higher at post assessment (*t* = − 2.28, *p* = .022, *d* = − 0.18), indicating a greater increase in classtime step counts over time in the experimental schools relative to the wait-list control schools. No significant group × time interactions were observed for step counts during recess, PE, or whole day (all Holm-Bonferroni adjusted *p*s ≥ .116).

For PA guideline adherence, no significant group × time interactions were observed for meeting the 30-min schooltime or 60-min daily MVPA recommendations (Holm-Bonferroni adjusted *p* = .622 and *p* = .972, respectively), indicating comparable declines across groups (see Table 2). Overall, PA recommendations were met more frequently for total daily MVPA than for school-based MVPA in both groups at both measurement points (all *p*s < .001).

### Exploratory analysis

**Fig. 4:**
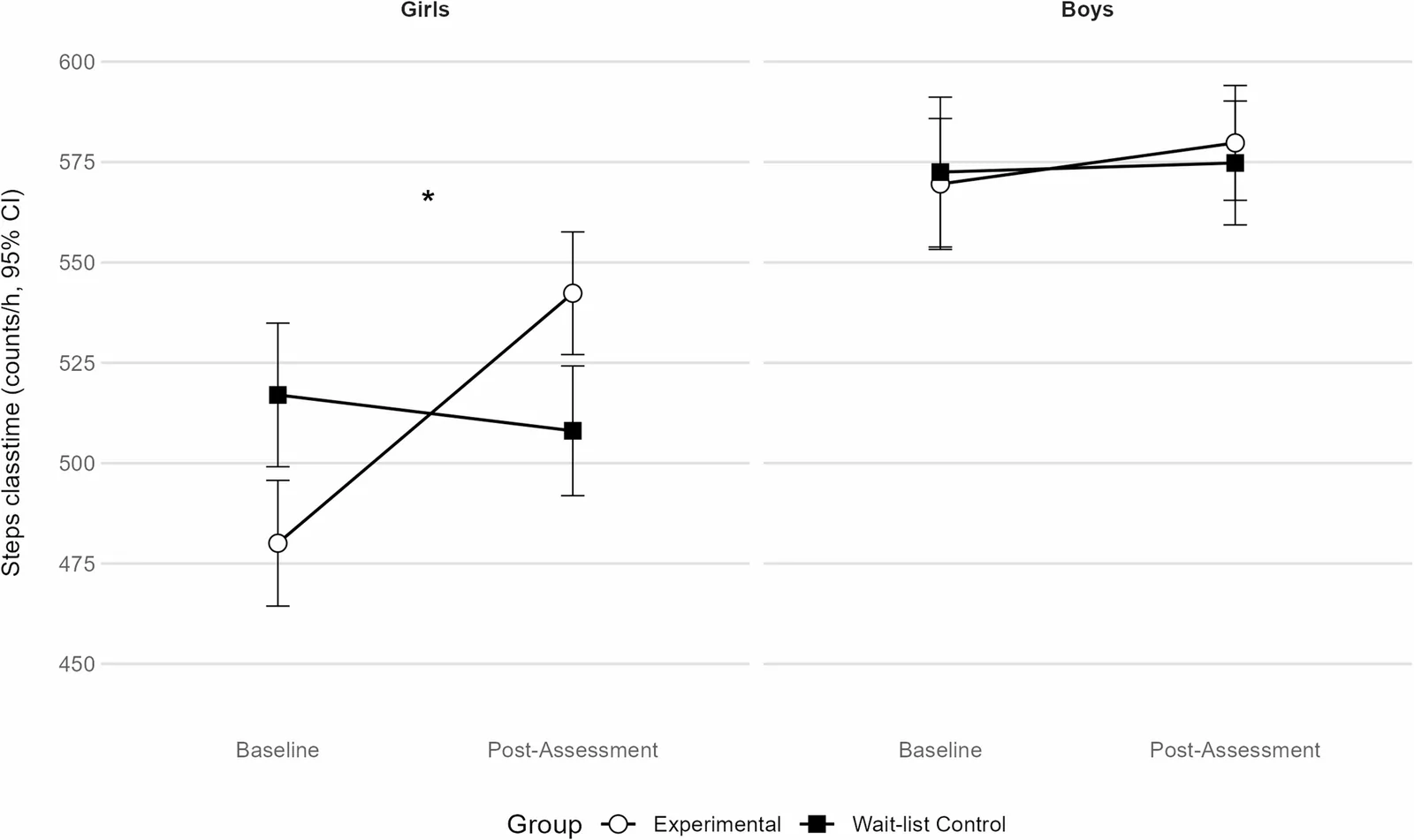
Mean steps during classtime (counts/h) at baseline and post assessment, stratified by group and gender. *adjusted *p* < .05

Gender-specific analyses revealed that across nearly all outcomes, no significant group × time × gender interactions were observed (all Holm–Bonferroni adjusted *p*s > .06). An overview of the descriptive statistics is provided in Table 3. An exception was classtime step counts, which showed a significant gender-specific intervention effect (*b* = 59.14, *SE* = 20.49, *t* = 2.89, Holm–Bonferroni adjusted *p* = .020, Cohen’s *d* = 0.50), driven by girls (see Fig. 4). Among girls, a significant group × time interaction was observed (*b* = 64.77, *SE* = 13.61, *t* = 4.75, *p* < .001, Cohen’s *d* = 0.56), reflecting a marked increase in step counts during classtime in experimental schools, whereas no such interaction was found among boys (*b* = 6.97, *SE* = 15.43, *t* = 0.82, *p* = .651, Cohen’s *d* = 0.06).

**Table 3:** Descriptive statistics (mean and SD) for primary and secondary PA outcomes at baseline and post assessment by study group

Subgroup		Experimental, Mean (SD)		Wait-list Control, Mean (SD)	
	Outcome	Baseline	Post	Baseline	Post
Boys	MVPA entire schooltime (%)	9.53 (2.93)	8.30 (2.30)	9.25 (2.75)	8.63 (2.66)
	MVPA classtime (%)	6.22 (2.22)	5.99 (1.84)	5.93 (2.30)	6.00 (2.28)
	MVPA recess (%)	22.76 (10.08)	17.44 (6.95)	19.74 (8.58)	16.54 (6.32)
	MVPA PE (%)	33.54 (10.08)	27.35 (9.21)	34.09 (11.45)	30.60 (10.69)
	MVPA WD (%)	10.21 (2.94)	8.85 (2.11)	9.60 (2.75)	9.04 (2.70)
	IN entire schooltime (%)	69.68 (5.27)	71.98 (4.43)	70.23 (5.24)	71.66 (4.76)
	IN classtime (%)	75.05 (4.77)	76.09 (4.07)	75.75 (4.93)	76.26 (4.53)
	IN recess (%)	43.57 (12.49)	50.99 (9.84)	46.24 (12.45)	50.78 (9.69)
	IN PE (%)	34.86 (12.35)	43.33 (12.73)	35.42 (14.61)	39.72 (14.28)
	IN WD (%)	69.95 (4.57)	71.83 (3.94)	70.90 (4.99)	71.56 (4.55)
	LPA entire schooltime (%)	20.87 (3.01)	19.67 (2.86)	20.49 (3.15)	19.65 (2.79)
	LPA classtime (%)	18.79 (3.08)	18.07 (2.81)	18.27 (3.15)	17.72 (2.87)
	LPA recess (%)	33.81 (5.64)	31.63 (4.71)	34.09 (6.55)	32.58 (5.37)
	LPA PE (%)	31.61 (5.39)	29.26 (5.87)	30.41 (6.92)	29.64 (5.87)
	LPA WD (%)	19.84 (2.55)	19.32 (2.56)	19.47 (2.90)	19.33 (2.63)
	Steps entire schooltime (counts/h)	719.04 (125.15)	698.74 (110.22)	728.76 (134.00)	704.51 (117.39)
	Steps classtime (counts/h)	569.54 (110.65)	579.77 (97.07)	572.50 (131.63)	574.77 (109.38)
	Steps recess (counts/h)	1613.38 (398.02)	1425.35 (290.82)	1567.24 (365.74)	1469.54 (374.01)
	Steps PE (counts/h)	1530.70 (311.84)	1426.10 (319.19)	1535.67 (340.00)	1462.11 (338.05)
	Steps whole day (count/h)	770.29 (133.88)	757.25 (107.77)	744.63 (136.68)	758.12 (126.97)
Girls	MVPA entire schooltime (%)	7.40 (2.40)	7.14 (2.16)	7.60 (2.48)	7.20 (2.31)
	MVPA classtime (%)	4.72 (1.83)	5.24 (1.83)	5.07 (2.11)	4.87 (2.04)
	MVPA recess (%)	16.74 (6.78)	13.78 (5.67)	14.84 (5.54)	13.79 (5.54)
	MVPA PE (%)	27.49 (9.75)	24.74 (9.48)	28.33 (9.02)	26.51 (8.94)
	MVPA WD (%)	8.75 (2.77)	8.25 (2.26)	8.24 (2.30)	7.98 (2.19)
	IN entire schooltime (%)	73.89 (5.08)	74.12 (4.34)	73.49 (5.19)	74.69 (4.72)
	IN classtime (%)	78.67 (4.60)	77.67 (4.16)	78.20 (5.04)	78.97 (4.63)
	IN recess (%)	51.87 (10.33)	55.65 (10.09)	52.85 (11.14)	55.82 (9.67)
	IN PE (%)	42.72 (14.62)	46.60 (14.38)	41.94 (11.92)	43.94 (12.45)
	IN WD (%)	71.60 (5.38)	72.17 (4.08)	71.77 (4.60)	72.67 (4.24)
	LPA entire schooltime (%)	18.69 (3.17)	18.77 (2.86)	18.89 (3.31)	18.11 (3.00)
	LPA classtime (%)	16.58 (3.13)	17.10 (2.96)	16.75 (3.37)	16.07 (3.05)
	LPA recess (%)	31.41 (6.19)	30.43 (6.20)	32.33 (7.53)	30.35 (5.79)
	LPA PE (%)	29.88 (6.79)	28.50 (6.85)	29.67 (5.83)	29.46 (6.39)
	LPA WD (%)	19.64 (3.27)	19.59 (2.63)	19.99 (2.99)	19.38 (2.79)
	Steps entire schooltime (counts/h)	615.40 (122.28)	649.09 (108.43)	657.33 (128.64)	637.43 (112.39)
	Steps classtime (counts/h)	480.06 (108.31)	542.32 (106.02)	517.00 (124.46)	508.06 (111.82)
	Steps recess (counts/h)	1426.23 (470.22)	1341.23 (431.59)	1441.08 (457.51)	1387.37 (444.74)
	Steps PE (counts/h)	1319.83 (351.93)	1339.83 (397.65)	1426.14 (329.88)	1407.14 (316.31)
	Steps whole day (count/h)	749.11 (156.20)	762.10 (136.62)	737.70 (120.47)	750.37 (113.36)

		**Experimental**, *n* **(%)**		**Wait-list Control**, *n* **(%)**	
	**Meeting PA - Recommendation**	**Baseline**	**Post**	**Baseline**	**Post**
Boys	30 min MVPA school time	111 (62.7)	80 (45.2)	111 (58.1)	94 (48.7)
	60 min MVPA whole day	165 (93.2)	161 (91.0)	173 (90.6)	171 (88.6)
Girls	30 min MVPA school time	65 (35.3)	57 (30.8)	67 (36.0)	61 (33.2)
	60 min MVPA whole day	152 (82.6)	149 (80.5)	150 (80.6)	143 (77.7)

*PA*  Physical activity, *PE*  Physical education, *WD*  Weekday, *MVPA*  Moderate-to-vigorous physical activity, *LPA*  Light physical activity, *IN*  Inactivity

Overall, boys exhibited higher activity levels than girls across most PA segments (all Holm–Bonferroni adjusted *p*s < .02), except for whole-day step counts at baseline (*p* = .183) and post assessment (*p* = .874). Consistently, boys were more likely to meet both school-based and daily PA recommendations at both time points (all *p*s < .05). Gender-specific descriptive statistics and test results are presented in Table S1 (Additional file 1).

**Fig. 5:**
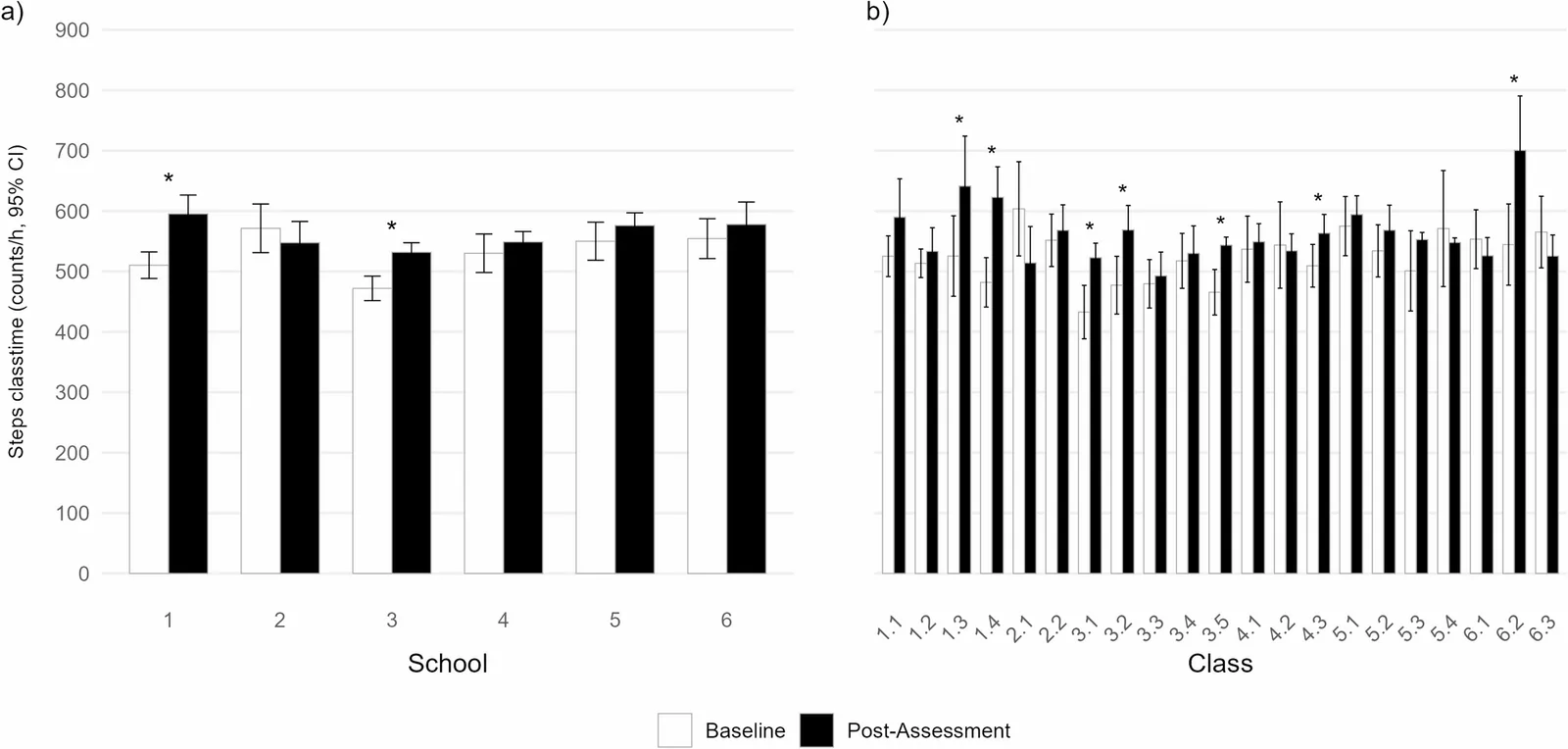
School- and class-level variation in mean steps during classtime (counts/h) in experimental schools only at baseline and post assessment. Numbers indicate (**a**) schools (1-6) and (**b**) classes nested within schools (e.g., 1.1–1.4). *indicates a significant within-unit pre–post difference (adjusted *p* < .032)

In addition, school- and class-level differences were explored for classtime step counts, the only outcome showing significant intervention effects. Differences between classes within schools accounted for 15.2% of the variance in change (ICC_class_ = 0.152), whereas variance between schools was small (ICC_school_ = 0.021); the remaining 82.7% was attributable to individual differences. Change scores differed significantly across classes, *F*(20, 327) = 4.19, *p* < .001, and across schools, *F*(5, 342) = 4.21, *p* = .001. Adjusted within-unit pre–post analyses identified significant increases in classtime step counts in two schools (Schools 1 and 3) and in seven classes (Classes 1.3, 1.4, 3.1, 3.2, 3.5, 4.3, and 6.2; all adjusted *p* < .023), highlighting substantial heterogeneity in intervention-related change (see Fig. 5).

## Discussion

This study evaluated the 12-month effectiveness of *Active School*, the first comprehensive PA program in Swiss secondary schools. The evaluation examined (1) changes in objectively measured PA across school segments and the whole day (2), effects on PA guideline adherence, and (3) exploratory analyses of gender-specific intervention effects and school- and class-level heterogeneity. No intervention effects were observed for the primary outcome (MVPA). Exploratory analyses showed increases in classtime step counts in experimental schools, particularly among girls, indicating segment- and gender-specific responsiveness. Exploratory analyses further revealed substantial class-level variability in intervention-related change.

### Segment-specific changes in PA

Worsening trends in MVPA, IN, or LPA were observed across the entire school time, recess, PE, and the whole day. Notably, MVPA during recess declined significantly more in the experimental schools than in the wait-list control schools. However, recess MVPA was higher in the experimental schools at baseline, whereas no group difference was observed at post assessment. This pattern suggests that the interaction may partly reflect regression to the mean rather than a genuine intervention effect [78]. Behavioral changes in the wait-list control schools during the study period may also have contributed to the observed findings. Because schools were recruited through self-volunteering following advertisement via newsletters and email invitation, selection bias cannot be excluded [79]. It is possible that schools choosing to participate differed systematically from non-participating schools, for example in their motivation, interest in physical activity promotion, or existing school health practices. Furthermore, the non-random allocation at the school level should be considered when interpreting the findings, as baseline differences between groups may reflect selection-related differences between schools, thereby increasing the risk of biased estimates and false-positive intervention effects. Overall, the observed decline in PA aligns with evidence from school-based PA interventions reporting limited or inconsistent effects on objectively measured PA, particularly during shorter implementation periods [20, 80]. However, these results contrast with reviews describing comprehensive school-based programs as among the most promising approaches for sustainable PA promotion [23], as well as with individual studies such as *PA4E* and *SCORES*, which demonstrated increases in PA intensity after 12 months and more pronounced effects after 24 months of implementation [28–30]. A central distinction between *Active School* and these programs is the degree of implementation autonomy. Whereas *PA4E* and *SCORES* implemented specified intervention strategies across predefined school segments, *Active School* explicitly granted schools flexibility to set tailored PA goals within selected segments based on internally led needs assessments [34]. This reflects a school-improvement logic, with evidence showing that such processes typically require several years before structural changes become routine and yield measurable outcomes [81]. Therefore, the 12-month evaluation likely captured an early implementation stage in which school-wide PA integration was possibly still developing and had not yet been implemented across the segments recess and PE, consistent with evidence suggesting that addressing a greater number of segments is associated with stronger outcomes [35]. However, no definitive conclusions can be drawn, as the present study reports outcome data only, while segment-specific implementation processes are being examined in a future mixed-methods evaluation [34].

An additional explanation for the absence of sustained effects across school segments, may lie in well-documented age-related decreases in PA during adolescence [82]. Such normative decreases appear particularly pronounced in less structured segments that rely heavily on students’ self-initiative, such as recess [43, 83], and may partly offset intervention-related gains. Even within PE, increasing curricular demands can constrain opportunities for sustained increases in physical activity intensity [84]. These developmental trends represent a persistent challenge for school-based PA promotion and may have contributed to the observed declines across segments despite ongoing implementation efforts.

Early implementation effects were most evident during classroom time, where MVPA remained stable while step counts increased in the experimental schools. However, because baseline classroom step counts were lower in the experimental schools, part of this increase may reflect regression to the mean rather than solely intervention-related effects [78]. Nevertheless, higher post-assessment step counts in the experimental schools still indicate a positive intervention effect. Overall, these findings may point to an initial focus on classroom-based strategies to enhance activity. Step-based metrics capture overall ambulatory volume independent of intensity thresholds [85]. They can reflect low-to-moderate activity changes not yet visible in MVPA outcomes and thus help detect early behavioral shifts, as suggested by studies showing divergent patterns of change in step counts and MVPA [86, 87]. The observed increase of ~ 40 steps per classtime hour corresponds to ~ 300–320 additional steps per school day. Although modest, such increments are meaningful, as higher daily step counts are consistently associated with more favorable indicators of body composition in children and adolescents, including BMI and body fat percentage [88]. This finding is particularly relevant as classtime represents the largest proportion of the school day. Furthermore, the early classtime effects are consistent with evidence from comprehensive school approaches, which show that classroom-based PA is commonly implemented first [43, 89, 90], as this structured setting allows PA to be embedded into established teaching routines under the direct guidance of teachers [91]. Accordingly, numerous studies have demonstrated that classtime-integrated PA approaches, such as active lessons or PA breaks, can yield short-term increases in PA [86, 92–94]. By contrast, unstructured school segments that rely more on student initiative, such as recess, may require greater autonomy-support, supervision, or environmental adaptations to promote active engagement [95, 96], suggesting that changes in these segments may manifest more slowly. However, it is assumed that as PA becomes a routine element of teaching practice, the broader school culture may begin to expect and support activity throughout the day [89]. As a practical implication, schools adopting a comprehensive school approach may consider using the classroom as an initial entry point for PA implementation, while simultaneously strengthening activity-promoting strategies in PE and especially in less structured segments such as recess [97].

### PA guideline adherence

*Active School* did not increase the proportion of students meeting either school-based or daily PA guidelines. Evidence on PA guideline compliance among adolescents remains scarce, and comprehensive approaches generally demonstrate null or only small effects on the proportion of participants achieving guidelines [29, 45, 98]. Notably, a high proportion of students in the present study already met the daily MVPA guideline at baseline (approximately 85–88%), which is substantially higher than reported in Swiss population-based data, where only 21.5% of adolescents met PA recommendations [2]. This discrepancy may partly reflect methodological differences, as previous studies used hip-worn ActiGraph devices, whereas the present study relied on wrist-worn GENEActiv monitors, which typically yield higher MVPA estimates [99]. In addition, these high baseline values may also have resulted in a ceiling effect, thereby limiting observable improvements in whole-day MVPA and overall guideline adherence [100]. Furthermore, the comparatively high baseline compliance may reflect selection bias at the school level, potentially resulting in a more physically active sample. Therefore, the representativeness and generalizability of the findings should be interpreted with caution. However, MVPA during school time at baseline remained low (approximately 8%) and comparable to previous Swiss studies [101]. Consequently, only around half of the study sample met the recommended 30 min of MVPA during school hours, highlighting the continued need for school-based PA promotion despite the high proportion of students meeting the overall daily PA recommendations.

Moreover, achieving improvements in guideline compliance likely requires explicit increases in activity intensity. Interventions specifically targeting high-intensity activity, such as HIIT-based programs, have shown clearer improvements in guideline adherence [102–104]. In the present study, early intervention effects were primarily observed for classtime step counts, suggesting initial implementation focused on PA volume rather than intensity, for example, through standing desks, short low-intensity PA breaks, or physically active learning [105]. From a health perspective, this underscores the importance of explicitly considering MVPA when defining PA-related goals within comprehensive school approaches [46]. While school autonomy and needs-based goal setting are essential for sustainability, early implementation phases may benefit from clearer guidance to ensure that selected strategies also target activity intensity. In practice, this may involve promoting sustained MVPA through structured and inclusive formats during recess, such as small-sided games, cooperative activities, or choice-based formats [106, 107]. In PE, this also includes optimizing lesson organization to minimize inactive time (e.g., reducing transition and waiting periods) and prioritizing continuous activity formats [108]. Additionally, short, high-activation PA breaks or dynamic learning tasks [92] within the classroom may further contribute to increasing MVPA across school segments.

Moreover, the high proportion of students meeting daily PA recommendations suggests that adolescents already accumulated a substantial share of their MVPA outside the school setting. This may indicate compensatory shifts in activity across contexts, potentially explaining the absence of intervention effects on physical activity intensity during school time. Relatively high MVPA levels during leisure time may have been offset by reduced activity during the school segments thereby limiting overall gains in total PA and school guideline adherence [109, 110]. Such patterns have been discussed in relation to the ActivityStat hypothesis [111], which proposes that children and adolescents may partially regulate total PA, such that increases in one context are accompanied by decreases in another.

### Gender-specific effects

Although boys were consistently more active than girls across all school segments, exploratory gender-stratified analyses showed that girls benefited more from the *Active School* intervention, as reflected by increased classtime step counts. This suggests meaningful gains for less active students, potentially reflecting gender-specific motivational orientations toward PA [47]. Girls may respond more favorably to structured and socially supportive activity formats, whereas boys often engage more readily in competitive and less structured settings [112–116]. These differences may partly explain the stronger classtime-related gains observed among girls [117].

However, the observed improvements were limited to step counts during classtime and did not extend to activity intensity, nor were comparable intervention effects observed among boys. Although boys were generally more active than girls, these findings underscore the importance of also addressing less active subgroups among boys, who may require different or more targeted strategies to elicit meaningful changes [118]. Such subgroups may include boys with overweight or low perceived competence in PA, who often experience motivational barriers and may benefit from non-competitive, autonomy-supportive approaches [119]. Rather than uniform PA promotion, results highlight identifying less active subgroups in girls and boys and tailoring strategies accordingly. Although student needs assessments were conducted in the present study to inform the selection of appropriate strategies, the findings suggest that gender-specific considerations may not have been sufficiently emphasized to better align intervention components with diverse motivational profiles [46, 49]. This underscores the central role of PALs, who are responsible for identifying students’ needs and shaping PA goals accordingly [34]. Future PAL training may therefore benefit from placing greater emphasis on gender-sensitive implementation strategies that support less active girls and boys [120].

### Heterogeneity of intervention-related changes across schools and classes

Exploratory analyses of classtime step counts in experimental schools—the only outcome showing significant intervention effects—revealed substantial heterogeneity across implementation units, driven primarily by differences between classes rather than schools. This pattern is consistent with previous research showing greater class-level than school-level variability in PA promotion, suggesting a stronger role of psychosocial than environmental factors [50, 51]. These findings suggest that teacher-related characteristics (e.g., motivation, competence) may contribute to implementation variability [27, 36, 52, 53] and should be examined in future process evaluations within *Active School* [34]. This suggests that future implementation may benefit from PALs providing targeted support to less engaged teachers, for example, through peer exchange or communities of practice [37], as well as to consider low-threshold, teacher-independent formats (e.g., video-based PA breaks) [121], to reduce implementation variability and improve effectiveness.

### Strengths, limitations, and future directions

This study has several strengths. First, the integrated PD training supported PALs as internal drivers of school-based PA promotion [37]. Second, PA was assessed objectively using GENEActiv accelerometers, providing valid, reliable data independent of self-report [65, 122]. Third, segment-specific analyses offered detailed insight into when and where PA promotion may be most effective [42].

However, at the present stage, the lack of detailed process data limits the interpretation of null effects and the understanding of mechanisms underlying variability across school segments [20]. Although the analyses indicate where changes occurred, it remains unclear which intervention components or implementation strategies were responsible for those changes. Future mixed-methods process evaluations incorporating implementation data and perspectives from students and teachers could help identify effective approaches and clarify whether intervention effects differ across individual characteristics and contextual factors, such as school segments or gender [38, 39]. A further key limitation lies in the voluntary participation of schools and the non-random allocation procedures, which may have introduced selection bias and limited the representativeness of the sample. In addition, constraints on randomization may have contributed to baseline differences between groups for some outcomes, thereby complicating the interpretation of intervention effects and increasing the risk of regression to the mean [123]. Moreover, the study was powered to detect overall intervention effects, but not subgroup effects; thus, gender-specific and heterogeneity findings should be interpreted as exploratory and hypothesis-generating. Furthermore, the sequential cohort design may have introduced cohort-specific influences that were not explicitly accounted for in the primary analyses. Finally, despite the use of high-quality accelerometer data, reduced wear compliance and substantial post assessment data loss posed challenges, as also reported in comparable studies [124]; simple reminder systems may help improve adherence in future research [29].

## Conclusion

This study evaluated the 12-month effectiveness of *Active School*, the first comprehensive PA promotion program in Swiss secondary schools. No effects on the primary outcome MVPA were observed. However, exploratory analyses showed increased classtime step counts in experimental schools, particularly among girls, suggesting that structured classroom-integrated activity may represent a potential approach for promoting PA within the school setting. Yet, the observed declines in MVPA indicate that all school-time segments may require more targeted and sustained implementation strategies. Mixed-methods process evaluations are needed to better understand implementation fidelity, contextual influences, and mechanisms underlying intervention heterogeneity. Overall, given the small intervention effects, uncertainty regarding implementation fidelity, and potential selection bias, as well as limited generalizability of the findings, the results should be interpreted with caution. Further research is needed to determine under which conditions *Active School* can be effectively and sustainably implemented in Swiss secondary schools.

## Supplementary Information

Supplementary Material 1. Additional file 1: This file includes a supplementary table on gender differences in PA outcomes at baseline and post assessment, detailed information on recruitment, sample representativeness, and missing data handling, as well as CONSORT and TIDieR checklists.

## Data Availability

The datasets used and analyzed during the current study are available from the corresponding author on reasonable request.

## Abbreviations

BMI: Body mass index
CSPAP: Comprehensive school physical activity program
ICC: Intraclass correlation coefficients
IN: Inactivity
LMM: Linear mixed effect models
LPA: Light physical activity
MVPA: Moderate-to-vigorous physical activity
PA: Physical activity
PAL: Physical activity leader
PD: Professional development
PE: Physical education
WHO: World Health Organization
