# Supplementary material for: Effectiveness of the Active School quasi-experimental cluster-controlled trial: Impact on objectively measured physical activity in Swiss secondary school students after 12 months
Source: BMC Public Health. 2026 Aug 5;26:3154. doi: 10.1186/s12889-026-28690-6 (PMC13725959; doi:10.1186/s12889-026-28690-6)
Supplement: Supplementary file 1 [file 12889_2026_28690_MOESM1_ESM.docx]

**Additional File 1**

This supplementary file provides additional information to support the interpretation and transparency of the study findings. It includes a supplementary table presenting gender differences in physical activity outcomes at baseline and post assessment. Furthermore, detailed information on sample recruitment, representativeness of the target population, differences between the recruited and analyzed samples, and the handling of missing data is provided, as required for empirical studies. In addition, the CONSORT checklist and the TIDieR checklist are included to document reporting quality and intervention description.

Table of Contents

[1 Table S1	2](#_Toc220392032)

[2 Supplementary Information on Sample Recruitment and Data Handling	3](#_Toc220392033)

[3 CONSORT Checklist	8](#_Toc220392034)

[4 TIDieR Checklist	11](#_Toc220392035)

# 1 Table S1

Table S1: Gender differences in primary and secondary physical activity outcomes and guideline adherence at baseline and post assessment

|  | **Baseline, Mean (SD)** | |  | **Gender Differences** | | |  | **Post Assessment, Mean (SD)** | |  | **Gender Differences** | | |
| --- | --- | --- | --- | --- | --- | --- | --- | --- | --- | --- | --- | --- | --- |
|  | **Boys** | **Girls** |  |  |  |  |  | **Boys** | **Girls** |  |  |  |  |
| **Measures** | (*n* = 370) | (*n* = 370) |  | *t* (738) | *p* | *d* |  | (*n* = 370) | (*n* = 370) |  | *t* (738) | *p* | *d* |
|  |  |  |  |  |  |  |  |  |  |  |  |  |  |
| **Primary Outcome PA** |  |  |  |  |  |  |  |  |  |  |  |  |  |
| MVPA entire schooltime (%) | 9.38 (2.84) | 7.50 (2.44) |  | 9.67 | < .001 | 0.71 |  | 8.44 (2.41) | 7.10 (2.13) |  | 7.32 | < .001 | 0.55 |
| MVPA classtime (%) | 6.07 (2.26) | 4.89 (1.98) |  | 7.54 | < .001 | 0.55 |  | 5.96 (2.03) | 4.99 (1.79) |  | 6.28 | < .001 | 0.46 |
| MVPA recess (%) | 21.19 (9.44) | 15.78 (6.25) |  | 9.16 | < .001 | 0.68 |  | 16.78 (6.33) | 13.67 (5.54) |  | 7.09 | < .001 | 0.52 |
| MVPA PE (%) | 33.83 (10.80) | 27.91 (9.39) |  | 7.90 | < .001 | 0.58 |  | 29.08 (9.89) | 25.70 (9.28) |  | 4.82 | < .001 | 0.35 |
| MVPA WD (%) | 9.89 (2.86) | 8.50 (2.56) |  | 7.04 | < .001 | 0.52 |  | 8.88 (2.31) | 8.06 (2.19) |  | 4.85 | < .001 | 0.36 |
|  |  |  |  |  |  |  |  |  |  |  |  |  |  |
| **Secondary Outcome PA** |  |  |  |  |  |  |  |  |  |  |  |  |  |
| IN entire schooltime (%) | 69.97 (5.25) | 73.69 (5.14) |  | -9.78 | < .001 | -0.72 |  | 71.82 (4.60) | 74.40 (4.54) |  | -7.69 | < .001 | -0.57 |
| IN classtime (%) | 75.42 (4.86) | 78.43 (4.83) |  | -8.42 | < .001 | -0.62 |  | 76.18 (4.31) | 78.32 (4.44) |  | -6.67 | < .001 | -0.49 |
| IN recess (%) | 44.95 (12.52) | 52.36 (10.74) |  | -8.58 | < .001 | -0.64 |  | 50.88 (9.75) | 55.74 (9.87) |  | -6.69 | < .001 | -0.49 |
| IN PE (%) | 35.15 (13.56) | 42.33 (13.32) |  | -7.24 | < .001 | -0.53 |  | 41.45 (13.67) | 45.27 (13.50) |  | -3.82 | < .001 | -0.28 |
| IN WD (%) | 70.44 (4.81) | 71.68 (5.00) |  | -3.48 | < .001 | -0.25 |  | 71.69 (4.27) | 72.42 (4.17) |  | -2.44 | 0.016 | -0.17 |
| LPA entire schooltime (%) | 20.67 (3.09) | 18.79 (3.24) |  | 8.00 | < .001 | 0.59 |  | 19.66 (2.82) | 18.44 (2.95) |  | 5.80 | < .001 | 0.42 |
| LPA classtime (%) | 18.52 (3.13) | 16.66 (3.25) |  | 7.85 | < .001 | 0.58 |  | 17.89 (2.85) | 16.59 (3.04) |  | 5.81 | < .001 | 0.44 |
| LPA recess (%) | 33.95 (6.13) | 31.87 (6.90) |  | 4.42 | < .001 | 0.32 |  | 32.13 (5.08) | 30.39 (5.99) |  | 4.38 | < .001 | 0.31 |
| LPA PE (%) | 30.99 (6.26) | 29.77 (6.32) |  | 2.57 | 0.020 | 0.19 |  | 29.46 (5.87) | 28.98 (6.63) |  | 1.03 | 0.606 | 0.08 |
| LPA WD (%) | 19.65 (2.74) | 19.82 (3.14) |  | -0.75 | 0.453 | -0.06 |  | 19.32 (2.60) | 19.48 (2.71) |  | -0.75 | 0.606 | -0.06 |
| Steps entire schooltime (counts/h) | 724.08 (129.73) | 636.48 (127.09) |  | 9.28 | < .001 | 0.68 |  | 701.75 (113.90) | 643.28 (110.43) |  | 7.06 | < .001 | 0.52 |
| Steps classtime (counts/h) | 571.08 (121.83) | 498.63 (118.01) |  | 8.21 | < .001 | 0.60 |  | 577.17 (103.56) | 525.24 (110.14) |  | 6.60 | < .001 | 0.49 |
| Steps recess (counts/h) | 1589.43 (381.78) | 1433.69 (463.30) |  | 4.99 | < .001 | 0.37 |  | 1448.40 (337.07) | 1364.24 (438.21) |  | 2.93 | 0.012 | 0.22 |
| Steps PE (counts/h) | 1533.28 (326.33) | 1373.28 (344.70) |  | 6.47 | < .001 | 0.48 |  | 1444.89 (329.22) | 1373.39 (360.49) |  | 2.82 | 0.015 | 0.21 |
| Steps whole day (counts/h) | 756.97 (135.77) | 743.37 (139.31) |  | 1.33 | 0.183 | 0.10 |  | 757.70 (118.02) | 756.25 (125.53) |  | 0.16 | 0.874 | 0.01 |
|  |  |  |  |  |  |  |  |  |  |  |  |  |  |
|  | **Baseline, n (%)** | |  | **Gender Differences** | | |  | **Post-Assessment, n (%)** | |  | **Gender Differences** | | |
|  | **Boys** | **Girls** |  | *X2(1)* | *p* | *V* |  | **Boys** | **Girls** |  | *X2(1)* | *p* | *V* |
| **Meeting PA-recommendation** |  |  |  |  |  |  |  |  |  |  |  |  |  |
| 30 min MVPA school | 222 (60.4) | 132 (35.6) |  | 45.89 | < .001 | 0.25 |  | 173 (46.8) | 117 (31.8) |  | 18.57 | < .001 | 0.16 |
| 60 min MVPA whole day | 338 (91.8) | 302 (81.6) |  | 16.75 | < .001 | 0.15 |  | 332 (89.7) | 292 (79.1) |  | 15.79 | < .001 | 0.15 |
|  |  |  |  |  |  |  |  |  |  |  |  |  |  |

Note: *p* values are Holm–Bonferroni adjusted for multiple comparisons. PA = physical activity; PE = physical education; WD = weekday; MVPA = moderate-to-vigorous physical activity; LPA = light physical activity; IN = inactivity, *d* = Cohen’s *d. V* = Cramer’s V.

# 2 Supplementary Information on Sample Recruitment and Data Handling

This chapter provides supplementary information on sampling procedures, representativeness of the analytical sample, differences between recruited and analyzed participants, and missing-data handling for the evaluation of *Active School*. It summarizes and complements methodological details reported in the main manuscript.

Schools were recruited via newsletters distributed through the Bern University of Teacher Education and through emails sent by the cantonal sports authority to all eligible secondary schools in the Canton of Bern. Eligible schools were public or semi-public secondary schools (grades 7–9) that did not specialize in specific learning needs and were not involved in other major physical activity promotion programs at the time of recruitment. Schools expressing interest were initially contacted for a meeting with the school principal, after which the research team provided an information session for the teaching staff. Final participation decisions were made internally within each school, and this direct personal contact proved valuable in facilitating recruitment. The study was conducted in two sequential cohorts. Each cohort comprised six schools. Within each cohort, schools were allocated to experimental (*n* = 3) and wait-list control (*n* = 3) conditions. Baseline assessments were conducted in summer 2023 (cohort 1) and summer 2024 (cohort 2), with post assessments occurring 12 months later. Allocation procedures were applied separately within each cohort. Therefore, the original study was designed as a cluster-randomized controlled trial; however, random allocation was partially constrained because three schools expressed strong interest in participating but were unable to begin implementation immediately and therefore joined the wait-list control condition. As a result, the evaluation followed a quasi-experimental cluster-controlled design, with baseline comparisons indicating no meaningful differences between experimental and wait-list control schools in key demographic variables relevant to the study. Given the nature of the school-based intervention, neither participants nor teaching staff could be blinded to group allocation. However, data analysts were blinded to condition during statistical analyses.

All 7th-grade students at participating schools were invited to take part, provided they were physically able to complete PA assessments and gave written parental consent. Prior to baseline data collection, the research team visited each participating class to explain the aims of the project and the upcoming assessment procedures, and to outline small incentives intended to support device wear-time compliance. Students subsequently received an information letter for their parents, including a consent form requiring parents to indicate whether their child was allowed to participate, as students were under the age of legal consent. Participation was voluntary and could be discontinued at any time without consequence. The study procedures, including the consent process, were approved by the Ethics Committee of the Faculty of Human Sciences at the University of Bern (Ref. 2023-03-05). Students who declined participation (*n* = 219, 23.75%) did so mainly for motivational or organizational reasons reported by teachers and parents. A total of 703 students consented and were assessed at baseline (345 students at experimental schools, 358 students at wait-list control schools). The sample represents a broad cross-section of secondary school students from the canton of Bern and aligns with typical characteristics of Swiss lower-secondary education in terms of age range, school structure and enrolment patterns. Although direct comparisons to population-wide reference values were not available for all indicators, available demographic characteristics suggest that the sample captures the diversity of the regional school population and reflects the target group for school-based PA promotion.

During the 12-month study period, data loss occurred due to accelerometer malfunction or invalid wear time (see data handling procedures below), school transfers, or voluntary withdrawal. Consistent with the longitudinal study design, an additional 39 students (21 from experimental schools and 18 from wait-list control schools) who either decided to participate in the study after baseline assessment or entered participating schools or classes during the study period were assessed at post assessment. In total, 600 students participated in the post assessment, resulting in an overall attrition of 103 students (14.7%) over the 12-month study period. For accelerometer-based physical activity outcomes, valid data were available for 606 of 703 students at baseline (86.2%) and for 428 of 600 students at post assessment (71.3%), corresponding to data loss rates of 13.8% and 28.7%, respectively. To clarify the handling of invalid accelerometer data, the following procedures were applied. Students were instructed to wear the accelerometer continuously (24 h/day) for nine consecutive days. To minimize potential reactivity effects, the first and last measurement days were excluded from the analyses. In addition, weekend days were excluded, as the primary focus of the present study was on school-related physical activity. Consequently, analyses were restricted to weekdays (school days) only, resulting in a maximum of five valid accelerometer days (Monday to Friday). A weekday was considered valid if participants accumulated at least 10 hours of wear time per day, excluding sleep (Antczak et al., 2021). Daily physical activity averages were calculated for participants with at least three valid schooldays, based exclusively on valid measurement days (Antczak et al., 2021). The original study protocol specified a minimum of four valid days to ensure reliable estimation of habitual physical activity across a full week. Previous reviews and methodological studies have suggested that a minimum of three weekdays and one weekend day provides sufficient reliability for estimating weekly physical activity patterns (Atkin et al., 2025; Rodrigues et al., 2025). As the present analyses focused exclusively on weekday (school days) activity and excluded weekend days, the criterion was adapted to require at least three valid weekdays, which was deemed appropriate for the present research question. For participants with fewer than three valid schooldays, average values were set to missing and subsequently imputed to preserve sample size and minimize bias associated with complete-case analyses. This approach is supported by previous methodological research demonstrating that imputation of missing and invalid accelerometer data yields more precise and less biased estimates than analyses based solely on observed data (Catellier et al., 2005). Missing values and cases at baseline and post-assessment were addressed using imputation. Little’s MCAR test was non-significant, indicating that the pattern of missingness did not deviate from missing completely at random. However, as the proportion of missing data exceeded 5% for key physical activity outcomes, multiple imputation with 10 imputed datasets was applied. Multiple imputation was performed in SPSS. Baseline and post assessment measures were jointly imputed within the same model. The imputation model included group (experimental vs. wait-list control), time (baseline, post), clustering variables (school, class, participant ID), and all covariates and outcome variables used in the main analyses. Prior to imputation, univariate outlier screening was conducted to identify implausible values indicative of potential measurement or data-entry errors; such cases were removed before the imputation procedure. The procedure resulted in an imputed study population of 742 students for baseline and post assessment analyses (365 students from experimental schools and 377 from wait-list control schools).

Analyses conducted separately in each imputed dataset and pooled using Rubin’s Rules to obtain final estimates. Complete-case sensitivity analyses yielded comparable findings, supporting the robustness of the imputed results. Attrition analyses showed no significant baseline differences between students who remained in the study and those who contributed data only at baseline with respect to sex, age, body mass index, or daily MVPA, indicating that dropout was unlikely to be selective in terms of key demographic and behavioral variables relevant to the research aims.

Taken together, recruitment procedures and attrition analyses indicate that the analytical sample provides an appropriate and transparent basis for evaluating the *Active School* program and for interpreting intervention-related changes in physical activity behavior.

Additional literature:

- Atkin AJ, Sherar LB, Ekelund U, Hansen BH, Andersen LB, Anderssen S, et al. Age-related change in children’s physical activity and sedentary time: The International Children’s Accelerometry Database (ICAD). *PLoS One*. 2025;20(9):e0327394. doi:10.1371/journal.pone.032739
- Antczak D, Lonsdale C, del Pozo Cruz B, Parker P, Sanders T. *Reliability of GENEActiv accelerometers to estimate sleep, physical activity, and sedentary time in children.* Int J Behav Nutr Phys Act. 2021;18(1):73. doi:10.1186/s12966-021-01143-6
- Catellier DJ, Hannan PJ, Murray DM, Addy CL, Conway TL, Yang S, et al. Imputation of missing data when measuring physical activity by accelerometry. *Med Sci Sports Exerc.* 2005;37(11 Suppl):555–562. doi:10.1249/01.mss.0000185655.38242.8e
- Rodrigues B, Videira-Silva A, Lopes L, Sousa-Sá E, Vale S, Cliff DP, et al. Methodological choices on 24-h movement behavior assessment by accelerometry: A scoping review. *Sports Med Open*. 2025;11:25. doi:10.1186/s40798-025-00820-1

# 3 CONSORT Checklist

|  | **Section/topic** | **No** | **CONSORT 2025 checklist item description** | **Reported on page no.** |
| --- | --- | --- | --- | --- |
|  | **Title and abstract** | | |  |
|  | Title and structured abstract | 1a | Identification as a randomised trial | 1 |
|  |  | 1b | Structured summary of the trial design, methods, results, and conclusions | 2-3 |
|  | **Open science** | | |  |
|  | Trial registration | 2 | Name of trial registry, identifying number (with URL) and date of registration | 3 |
|  | Protocol and statistical analysis plan | 3 | Where the trial protocol and statistical analysis plan can be accessed | 10 |
|  | Data sharing | 4 | Where and how the individual de-identified participant data (including data dictionary), statistical code and any other materials can be accessed | 30 |
|  | Funding and conflicts of interest | 5a | Sources of funding and other support (eg, supply of drugs), and role of funders in the design, conduct, analysis and reporting of the trial | 30 |
|  |  | 5b | Financial and other conflicts of interest of the manuscript authors | 30 |
|  | **Introduction** | | |  |
|  | Background and rationale | 6 | Scientific background and rationale | 4-6 |
|  | Objectives | 7 | Specific objectives related to benefits and harms | 6 |
|  | **Methods** | | |  |
|  | Patient and public involvement | 8 | Details of patient or public involvement in the design, conduct and reporting of the trial | 6-7 |
|  | Trial design | 9 | Description of trial design including type of trial (eg, parallel group, crossover), allocation ratio, and framework (eg, superiority, equivalence, non-inferiority, exploratory) | 6-7 |
|  | Changes to trial protocol | 10 | Important changes to the trial after it commenced including any outcomes or analyses that were not prespecified, with reason | 6-7 |
|  | Trial setting | 11 | Settings (eg, community, hospital) and locations (eg, countries, sites) where the trial was conducted | 6-7 |
|  | Eligibility criteria | 12a | Eligibility criteria for participants | 7 |
|  |  | 12b | If applicable, eligibility criteria for sites and for individuals delivering the interventions (eg, surgeons, physiotherapists) | - |
|  | Intervention and comparator | 13 | Intervention and comparator with sufficient details to allow replication. If relevant, where additional materials describing the intervention and comparator (eg, intervention manual) can be accessed | 10 |
|  | Outcomes | 14 | Prespecified primary and secondary outcomes, including the specific measurement variable (eg, systolic blood pressure), analysis metric (eg, change from baseline, final value, time to event), method of aggregation (eg, median, proportion), and time point for each outcome | 11-13 |
|  | Harms | 15 | How harms were defined and assessed (eg, systematically, non-systematically) | - |
|  | Sample size | 16a | How sample size was determined, including all assumptions supporting the sample size calculation | 7 |
|  |  | 16b | Explanation of any interim analyses and stopping guidelines | 13, additional file 1 |
|  | Randomisation: |  |  |  |
|  | Sequence generation | 17a | Who generated the random allocation sequence and the method used | 6, additional file 1 |
|  |  | 17b | Type of randomisation and details of any restriction (eg, stratification, blocking and block size) | 6, additional file 1 |
|  |  |  |  | **Reported on page no.** |
|  | Allocation concealment mechanism | 18 | Mechanism used to implement the random allocation sequence (eg, central computer/telephone; sequentially numbered, opaque, sealed containers), describing any steps to conceal the sequence until interventions were assigned | 6-7, additional file 1 |
|  | Implementation | 19 | Whether the personnel who enrolled and those who assigned participants to the interventions had access to the random allocation sequence | Additional file 1 |
|  | Blinding | 20a | Who was blinded after assignment to interventions (eg, participants, care providers, outcome assessors, data analysts) | Additional file 1 |
|  |  | 20b | If blinded, how blinding was achieved and description of the similarity of interventions | Additional file 1 |
|  | Statistical methods | 21a | Statistical methods used to compare groups for primary and secondary outcomes, including harms | 13, 14 |
|  |  | 21b | Definition of who is included in each analysis (eg, all randomised participants), and in which group | 9, additional file 1 |
|  |  | 21c | How missing data were handled in the analysis | 8, additional file 1 |
|  |  | 21d | Methods for any additional analyses (eg, subgroup and sensitivity analyses), distinguishing prespecified from post hoc | 13 |
|  | **Results** | | |  |
|  | Participant flow, including flow diagram | 22a | For each group, the numbers of participants who were randomly assigned, received intended intervention, and were analysed for the primary outcome | 9, 13-19 |
|  |  | 22b | For each group, losses and exclusions after randomisation, together with reasons | additional file 1 |
|  | Recruitment | 23a | Dates defining the periods of recruitment and follow-up for outcomes of benefits and harms | 11 |
|  |  | 23b | If relevant, why the trial ended or was stopped | - |
|  | Intervention and comparator delivery | 24a | Intervention and comparator as they were actually administered (eg, where appropriate, who delivered the intervention/comparator, how participants adhered, whether they were delivered as intended (fidelity)) | 10-11 |
|  |  | 24b | Concomitant care received during the trial for each group | 10-11 |
|  | Baseline data | 25 | A table showing baseline demographic and clinical characteristics for each group | 15 |
|  | Numbers analysed,  outcomes and estimation | 26 | For each primary and secondary outcome, by group:  ● the number of participants included in the analysis  ● the number of participants with available data at the outcome time point  ● result for each group, and the estimated effect size and its precision (such as 95% confidence interval)  ● for binary outcomes, presentation of both absolute and relative effect size | 17-18 |
|  | Harms | 27 | All harms or unintended events in each group | 7, 8 |
|  | Ancillary analyses | 28 | Any other analyses performed, including subgroup and sensitivity analyses, distinguishing pre-specified from post hoc | 18-21, additional file 1 |
|  | **Discussion** | | |  |
|  | Interpretation | 29 | Interpretation consistent with results, balancing benefits and harms, and considering other relevant evidence | 21-29 |
|  | Limitations | 30 | Trial limitations, addressing sources of potential bias, imprecision, generalisability, and, if relevant, multiplicity of analyses | 28 |

Citation: Hopewell S, Chan AW, Collins GS, Hróbjartsson A, Moher D, Schulz KF, et al. CONSORT 2025 Statement: updated guideline for reporting randomised trials. BMJ. 2025; 388:e081123. <https://dx.doi.org/10.1136/bmj-2024-081123>
© 2025 Hopewell et al. This is an Open Access article distributed under the terms of the Creative Commons Attribution License (<https://creativecommons.org/licenses/by/4.0/>), which permits unrestricted use, distribution, and reproduction in any medium, provided the original work is properly cited.

*We strongly recommend reading this statement in conjunction with the CONSORT 2025 Explanation and Elaboration and/or the CONSORT 2025 Expanded Checklist for important clarifications on all the items. We also recommend reading relevant CONSORT extensions. See [www.consort-spirit.org](http://www.consort-spirit.org).

# 4 TIDieR Checklist

| **Item number** | **Item** | **Where located **** | |
| --- | --- | --- | --- |
|  |  | Primary paper  (page or appendix  number) | Other ^†^ (details) |
|  | **BRIEF NAME** |  |  |
| **1.** | Provide the name or a phrase that describes the intervention. | 5/10 | ______________ |
|  | **WHY** |  |  |
| **2.** | Describe any rationale, theory, or goal of the elements essential to the intervention. | 10 | Study protocol |
|  | **WHAT** |  |  |
| **3.** | Materials: Describe any physical or informational materials used in the intervention, including those provided to participants or used in intervention delivery or in training of intervention providers. Provide information on where the materials can be accessed (e.g. online appendix, URL). | 10 | Study protocol |
| **4.** | Procedures: Describe each of the procedures, activities, and/or processes used in the intervention, including any enabling or support activities. | 5, 10 | Study protocol |
|  | **WHO PROVIDED** |  |  |
| **5.** | For each category of intervention provider (e.g. psychologist, nursing assistant), describe their expertise, background and any specific training given. | 5, 10 | Study protocol |
|  | **HOW** |  |  |
| **6.** | Describe the modes of delivery (e.g. face-to-face or by some other mechanism, such as internet or telephone) of the intervention and whether it was provided individually or in a group. | 10 | Study protocol |
|  | **WHERE** |  |  |
| **7.** | Describe the type(s) of location(s) where the intervention occurred, including any necessary infrastructure or relevant features. | 10 | Study protocol |
|  | **WHEN and HOW MUCH** |  |  |
| **8.** | Describe the number of times the intervention was delivered and over what period of time including the number of sessions, their schedule, and their duration, intensity or dose. | 10, 11 | Study protocol |
|  | **TAILORING** |  |  |
| **9.** | If the intervention was planned to be personalised, titrated or adapted, then describe what, why, when, and how. | 10 | Study protocol |
|  | **MODIFICATIONS** |  |  |
| **10.^ǂ^** | If the intervention was modified during the course of the study, describe the changes (what, why, when, and how). | - | - |
|  | **HOW WELL** |  |  |
| **11.** | Planned: If intervention adherence or fidelity was assessed, describe how and by whom, and if any strategies were used to maintain or improve fidelity, describe them. | 28 | Study protocol |
| **12.^ǂ^** | Actual: If intervention adherence or fidelity was assessed, describe the extent to which the intervention was delivered as planned. | - | - |

** **Authors** - use N/A if an item is not applicable for the intervention being described. **Reviewers** – use ‘?’ if information about the element is not reported/not sufficiently reported.

† If the information is not provided in the primary paper, give details of where this information is available. This may include locations such as a published protocol or other published papers (provide citation details) or a website (provide the URL).

ǂ If completing the TIDieR checklist for a protocol, these items are not relevant to the protocol and cannot be described until the study is complete.

* We strongly recommend using this checklist in conjunction with the TIDieR guide (see *BMJ* 2014;348:g1687) which contains an explanation and elaboration for each item.

* The focus of TIDieR is on reporting details of the intervention elements (and where relevant, comparison elements) of a study. Other elements and methodological features of studies are covered by other reporting statements and checklists and have not been duplicated as part of the TIDieR checklist. When a **randomised trial** is being reported, the TIDieR checklist should be used in conjunction with the CONSORT statement (see [www.consort-statement.org](http://www.consort-statement.org)) as an extension of **Item 5 of the CONSORT 2010 Statement.** When a **clinical trial** **protocol** is being reported, the TIDieR checklist should be used in conjunction with the SPIRIT statement as an extension of **Item 11 of the SPIRIT 2013 Statement** (see [www.spirit-statement.org](http://www.spirit-statement.org)). For alternate study designs, TIDieR can be used in conjunction with the appropriate checklist for that study design (see [www.equator-network.org](http://www.equator-network.org)).
